# A rare case of a patient with resistant schizophrenia who hears a voice reading the texts instead of being read in her mind

**DOI:** 10.1192/bjo.2021.344

**Published:** 2021-06-18

**Authors:** Thilini Nanayakkara, Vipula Wijesiri

**Affiliations:** Teaching Hospital Peradeniya

## Abstract

**Objective:**

This case is presented to describe a rare psychopathology in which the patient hears her own voice speaking out loud all the texts that she sees in books or papers and she cannot read them inside her mind. This psychopathological phenomena has some features of reflex hallucinations, thought echo as well as of inner reading voices yet it cannot be categorized into either one.

**Case report:**

This is a 26-year-old female with Schizophrenia for 3 years. While on medication 8 months before presentation she started hearing her own voice reading any text that she sees. When she sees a text she cannot read it in her mind and understand, but she hears it in her own voice to her ears. With this she also hears other voices talking about her and to her. She also believes that her father is the one who controls all her actions and the things that happen to her. In her mental state examination her mood was euthymic and she had delusions of control, thought broadcasting and in her perceptions she had visual perceptual abnormality where she saw the same object she would look at in another direction but they are under her control. She also had second and third person auditory hallucinations. She was admitted to start on clozapine because her voices did not respond to any medication.

**Discussion:**

Auditory hallucinations are the most commonly encountered type in schizophrenia with a prevalance of 70–80%. This patient hears the words that she sees which has some features of reflex hallucinations, however in the latter the hallucination is not a transformation of the perception. This also has some qualities of thought echo, where just as the patient thinks she can hear them. However in this patient she cannot read the texts in her mind. Inner reading voices are where a person talks to oneself while reading, however in the subjective mind. In our patient this phenomenon also proved to be the most difficult to treat as all her other auditory hallucinations responded to Clozapine, while still this phenomenon remained.

**Conclusion:**

This case is presented to describe the rare psychopathology in this patient in the form of auditory hallucinations

